# Efficacy of catheter‐based drug delivery in a hybrid in vitro model of cardiac microvascular obstruction with porcine microthrombi

**DOI:** 10.1002/btm2.10631

**Published:** 2023-12-08

**Authors:** Yannick Rösch, Thorald Stolte, Miriam Weisskopf, Sabrina Frey, Rob Schwartz, Nikola Cesarovic, Dominik Obrist

**Affiliations:** ^1^ ARTORG Center for Biomedical Engineering Research University of Bern Bern Switzerland; ^2^ Department of Health Science and Technology ETH Zurich Zurich Switzerland; ^3^ Center for Preclinical Development University Hospital Zurich, University of Zurich Zurich Switzerland; ^4^ CorFlow Therapeutics AG Baar Switzerland; ^5^ Department of Cardiothoracic and Vascular Surgery Deutsches Herzzentrum der Charité (DHZC) Berlin Germany

**Keywords:** coronary circulation, intracoronary controlled flow infusion, microfluidic chip, microvascular obstruction, proximal balloon occlusion

## Abstract

Microvascular obstruction (MVO) often occurs in ST‐elevation myocardial infarction (STEMI) patients after percutaneous coronary intervention (PCI). Diagnosis and treatment of MVO lack appropriate and established procedures. This study focused on two major points by using an in vitro multiscale flow model, which comprised an aortic root model with physiological blood flow and a microfluidic model of the microcirculation with vessel diameters down to 50 μm. First, the influence of porcine microthrombi (MT), injected into the fluidic microchip, on perfusion was investigated. We found that only 43% of all injected MT were fully occlusive. Second, it could also be shown that the maximal concentration of a dye (representing therapeutic agent) during intracoronary infusion could be increased on average by 58%, when proximally occluding the coronary artery by a balloon during drug infusion. The obtained results and insights enhance the understanding of perfusion in MVO‐affected microcirculation and could lead to improved treatment methods for MVO patients.

**Translational Impact Statement**

This in vitro study provides information on the distribution and behavior of embolizing microthrombi in an microvascular obstruction‐affected coronary network, and shows the benefits of proximal intracoronary balloon occlusion, which significantly increases the efficacy of intracoronary drug infusion and, thereby, could improve the treatment of ST‐elevation myocardial infarction patients.

## 1 INTRODUCTION

Cardiac microvascular obstruction (MVO) occurs in 40%–60% of patients with ST‐elevation myocardial infarction (STEMI), despite previous successful percutaneous coronary intervention (PCI).^1^, ^2^, ^3^ MVO has several causes: residual particles of the primary thrombus washed into the microcirculation (microthrombi [MT]), myocardial tissue and endothelial cell swelling, and collapsed vessels in the coronary microcirculation. MVO leads to underperfused or nonperfused regions of the myocardium^4^, ^5^, ^6^, ^7^, ^8^ and has a substantial negative prognostic impact.^1^, ^3^, ^9^

However, MVO cannot be detected during acute STEMI treatment due to the limited resolution of fluoroscopic angiography.^10^ Today's gold standard for MVO diagnosis is based on detecting nonperfused regions using late‐gadolinium‐enhanced cardiac magnetic resonance imaging (MRI).^11^ MRI, however, is impractical or even impossible in acute diagnosis and treatment of STEMI patients.

Even if MVO is diagnosed, the outcome is often nonsatisfactory because no established treatment exists. Direct microvascular catheter access is impossible due to the affected vessel's size and the extensive microvascular network.^4^ Systemic administration of therapeutic agents has limited or no efficacy, and direct intracoronary drug infusion shows no significant advantage over intravenous delivery.^12^, ^13^, ^14^, ^15^

In Rösch et al.,^16^ a multiscale in vitro benchtop model was presented. The large scales of the model consisted of a left heart mock loop, driven by a computer‐controlled pump, that created physiological blood flow in a silicone casted aortic root phantom. In a coronary model (mid‐scale), the flow conditions in the left anterior descending (LAD) coronary artery were reproduced. This model was connected to an ostium in the silicone aortic root phantom and consisted of several resistor and compliance elements in a serial layout. The large and mid‐scale of the model were tuned to reproduce the coronary hemodynamics of a porcine animal model with MVO. To this end, data from the porcine model was previously obtained by an occlusion‐infusion sequence (OIS) with the controlled flow infusion (CoFI) system (CorFlow therapeutics AG, Baar, Switzerland; Figure 6d).^17^

To mimic the myocardial microcirculation of the coronary system (small scale), a selective laser etched (SLE) microfluidic chip (microchip) contained channels with diameters between 555 and 50 μm, and represents two coronary arterial vessels of order‐8 including all daughter vessels down to order‐5 (based on morphological data proposed in Kassab et al.^18^ and Kalsho et al.^19^).

**FIGURE 1:**
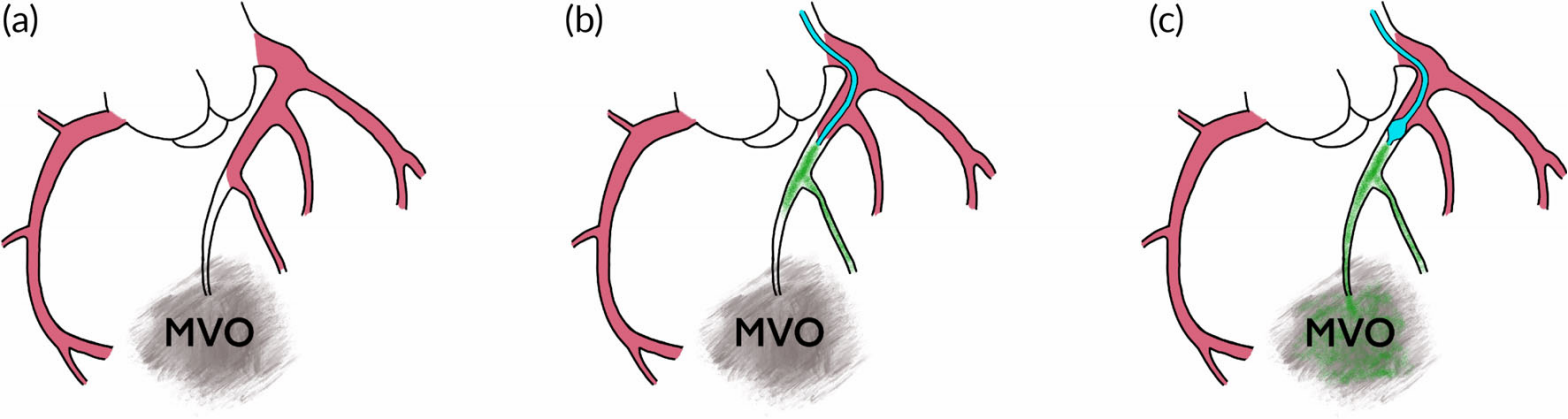
(a) Schematic of an microvascular obstruction (MVO) region with poor perfusion. (b) Treatment of MVO with an intracoronary infusion. Most of the drug is washed out through nonoccluded vessels. (c) Treatment of MVO with balloon‐occlusion infusion leads to enhanced drug delivery toward the MVO (adapted Figure from Ref. 16).

This multiscale in vitro setup of the coronary system was used to show that the efficacy of drug delivery into MVO‐affected regions of an arteriolar tree correlates with the impaired perfusion of these regions, which may explain the limited success of treatments with pharmaceutics infused in the proximal bloodstream. To this end, two infusion cases have been tested: the *balloon* case and *the no‐*balloon case. Like this, it was shown that using a multi‐lumen occlusion‐infusion catheter can increase the local drug concentration in MVO regions: in the *balloon* case, a balloon was inflated proximal to the MVO region, so that blood flow to the affected vessels was temporarily blocked. Through a second catheter lumen with an infusion opening distal to the balloon (Figure 6c), 15 mL dye, modeling a drug suspension, was infused in 30s. The balloon remained inflated for 60s after the infusion ended (holding phase). During the whole experiment, the distribution of the dye in the fluidic microchip was recorded. Compared with a catheter‐based infusion without balloon (*no‐balloon* case), the maximal dye concentration in MVO‐affected vessels could be increased by a factor of 2.2–3.2. The basic concept of this method is illustrated in Figure 1.

However, in these experiments, MVO was modeled simply by blocking the outlets on one side of the microchip. Although this reproduced the hemodynamic condition in an MVO‐affected region, it did not accurately reproduce the situation in the immediate neighborhood of an actual obstruction. Previous studies reported that obstructions are situated in vessels smaller than 120μm.^4^ This study addresses this limitation. It is based on the same setup and treatment method as in Rösch et al.^16^ but uses an improved MVO model using real porcine arterial MT that are randomly injected and deposited into the used microchip such that complex configurations of entirely blocking and semipermeable obstructions at different levels of the microvascular tree can be studied. This report analyses how these MT can affect an arteriolar network. It also shows that the used occlusion‐infusion catheter can help increasing the local drug concentration close to the occluding MT.

## 2 RESULTS

### 2.1 Microthrombi were more frequent in channels <250 μm and at bifurcations

**FIGURE 2:**
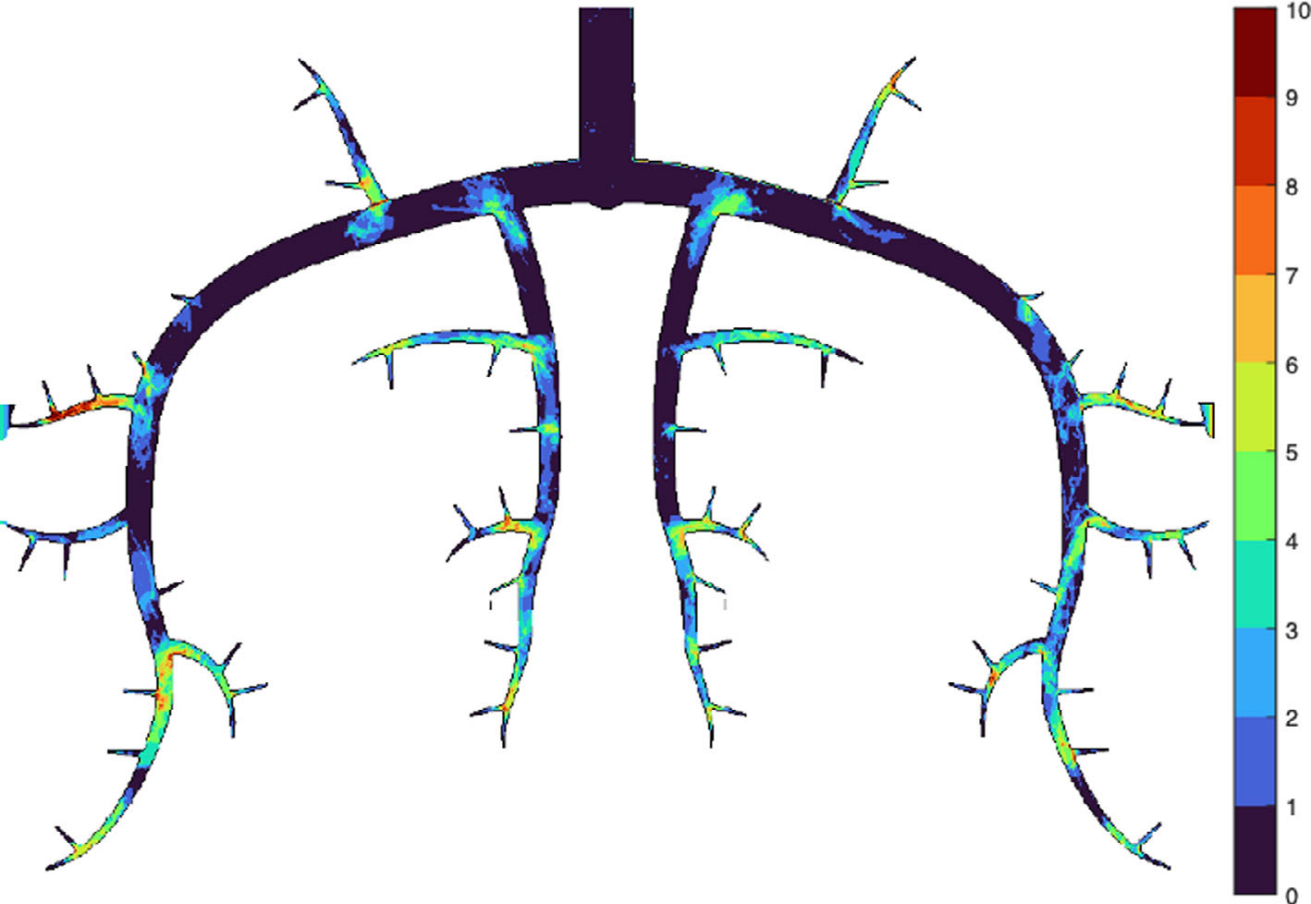
Heatmap of the microchip showing where all the 282 microthrombi resided in the microchip.

**FIGURE 3:**
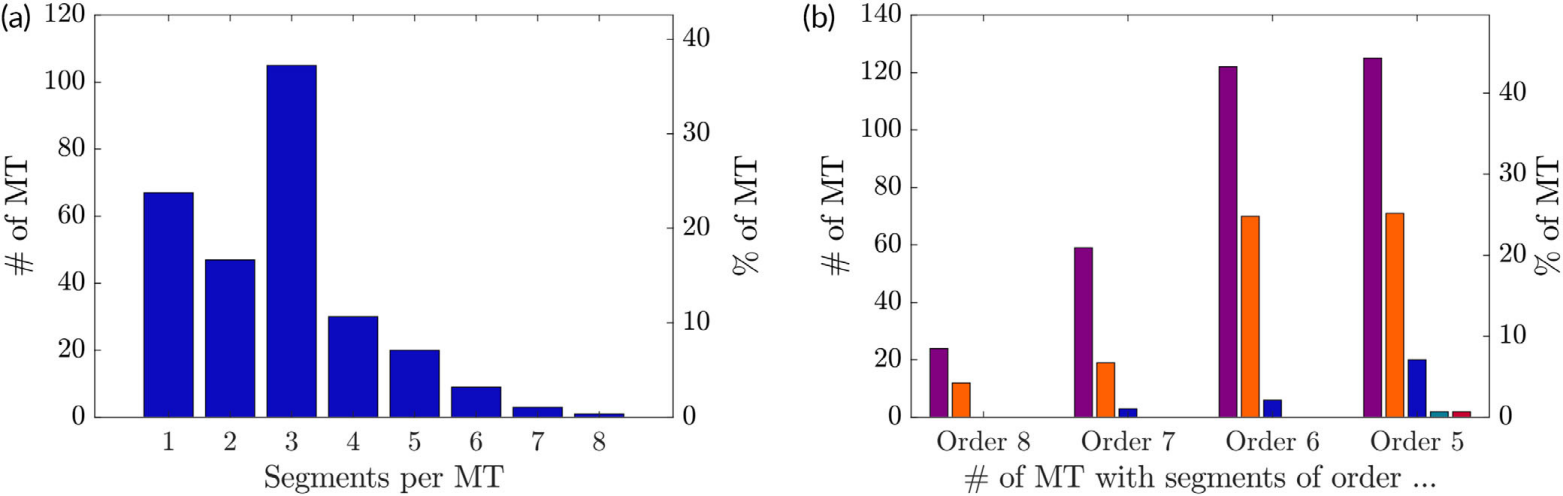
(a) Size of microthrombi (MT) by number of thrombus segments. (b) Number of thrombus segments of same order occupied by the same microthrombus (purple: 1 segment, orange: 2 segments, blue: 3 segments, petrol: 4 segments, red: 5 segments).

Over the course of 20 independent experiments (MVO settings), a total of 282 MT was detected. Figure 2 shows a heatmap indicating how often an MT was detected at a particular location in the microchip. The distribution of MT appears very symmetrical, that is, there does not seem to be a bias toward either side of the microchip. In general, more MT were detected in the smaller and more distal channels. In the larger channels, more MT were found close to bifurcations. Parts of these MT typically also penetrated the smaller adjacent channel of the respective bifurcation. Figure 3a illustrates that 21% of all MT resided just in one single segment, whereas 37% penetrated simultaneously three different vessel segments. Over 40% of all MT were located in at least one vascular segment of order 5 (100–50 μm) or order 6 (≈200–100 μm), while 25% of all MT covered at least two order 5 or two order 6 thrombus‐segments (Figure 3b).

Further, the area occupied by the MTs was analyzed (Figure S1). The mean projected area of the MTs was 0.11 mm^2^ (median: 0.07 mm^2^, 25%‐quartile: 0.03 mm^2^, 75%‐quartile: 0.15 mm^2^).

### 2.2 More than 75% of microthrombi could be reached by drug

**FIGURE 4:**
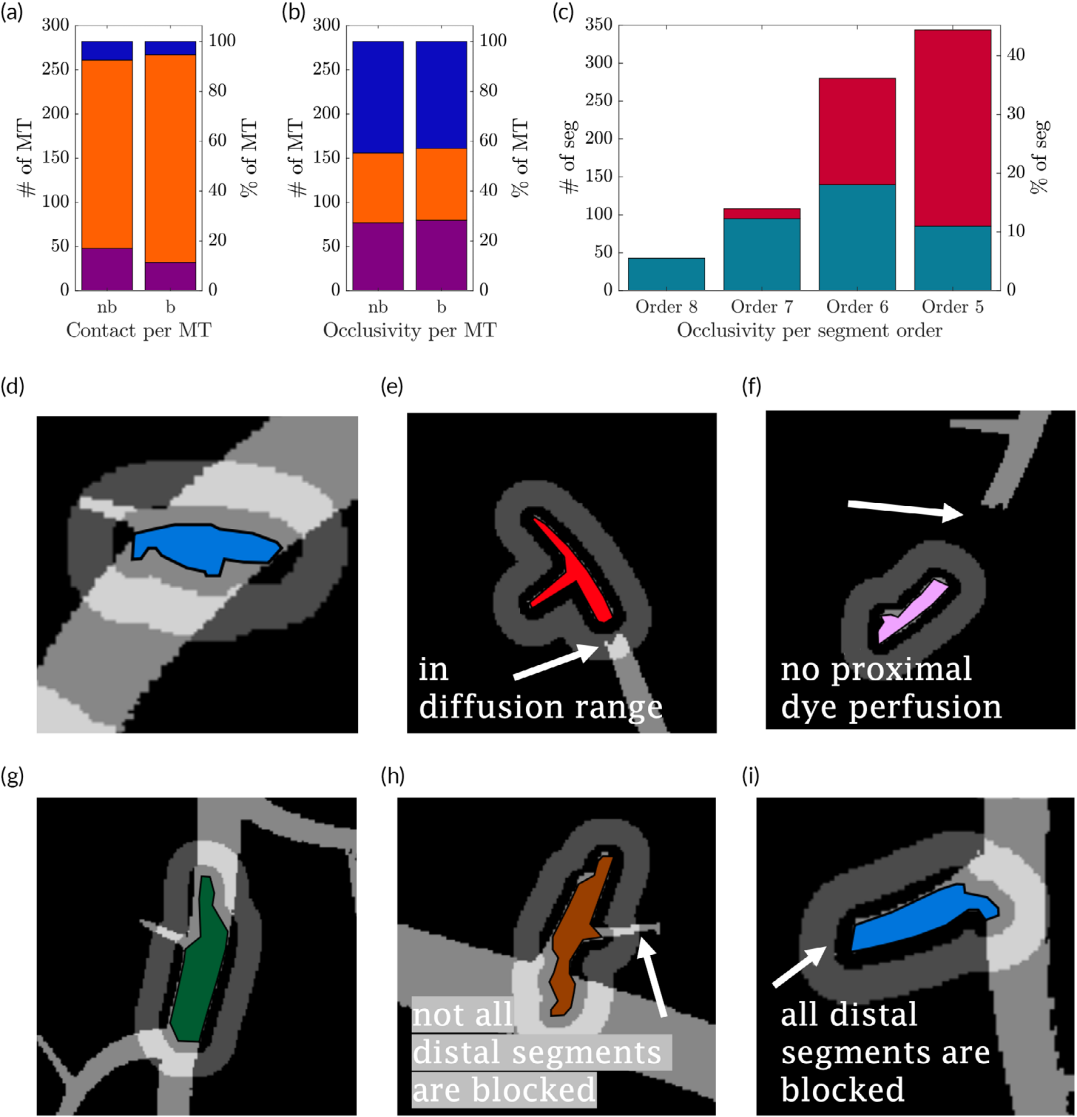
(a) Number of microthrombi (MT) that have no contact with the dye (purple), had contact (orange) and were in diffusion range (blue). (b) Number of MT that were nonocclusive (purple), semi‐occlusive (orange) and were fully occlusive (blue). (c) Number of thrombus segments, sorted by order, that were nonocclusive (petrol) or were fully occlusive (red). Forms of occlusivity (d‐f) and contact (g,h): (d) nonocclusive, (e) semiocclusive, (f) fully occlusive, (g) in contact, (h) diffusion range, and (i) no‐contact. Light gray shows regions in the microchip where the maximum dye concentration is higher than 20%. The darker gray annulus around the colored MT is the defined region of interest to evaluate the concentration in close proximity to the MT.

Every microthrombus was categorized according to the concentration level in a surrounding annulus‐shaped region of interest (ROI) with a width of 240 μm. They were considered to be in *contact* the concentration was higher than 20% at the edge of the thrombus. They were in the *diffusion range* if 20% concentration was reached only in the annulus, or they were in *no‐contact* if the maximum concentration was <20% in the whole ROI. Figure 4d–f shows examples of all three types of contact.

Figure 4a shows that most of the MT (79%) had *contact* with the drug, and only (14%) had *no‐contact* at all in the balloon case. In the *no‐balloon* case, 75% were in contact, and 17% had *no‐contact*.

Figure 4a also shows that, as for the kind of occlusivity, there is almost no difference between the *no‐balloon* (*nb*) and the *balloon* (*b*) case regarding the type of contact.

### 2.3 Less than 50% of microthrombi were fully occlusive

Three different types of flow obstruction caused by the MT could be discriminated. All vascular segments of the chip that were penetrated by a specific MT (thrombus segments) were classified as *nonocclusive* or *occlusive*, depending on the maximum dye concentration during the infusion protocol (concentration threshold of 20%). Then, the MTs were classified as *nonocclusive* (all thrombus segments were *nonocclusive*), *fully occlusive* (all thrombus segments were occlusive), or *semiocclusive* (otherwise). Figure 4g–i shows examples of all three kinds of occlusivity.

Figure 4b also shows that 29% of the MT were *semiocclusive*, and 43% were *fully occlusive* in the *balloon* case (no‐balloon: 28% semiocclusive, 45% fully occlusive). Therefore, the proximal inflation of the balloon during infusion (*balloon* vs. *no‐balloon* case from Ref. 16) had almost no effect on the kind of occlusivity (Figure 4b). Note that for both protocols, the same amount of dye was infused (15 mL in 30 s), and that they differed only in the inflation of the balloon and the following holding phase.

In Figure 4c, the occlusivity of single thrombus segments is described. The lower the order of the channel, the more they were affected by thrombi and the more often they were *occlusive*.

### 2.4 The presence of microthrombi decreased drug availability at target by 34%

**FIGURE 5:**
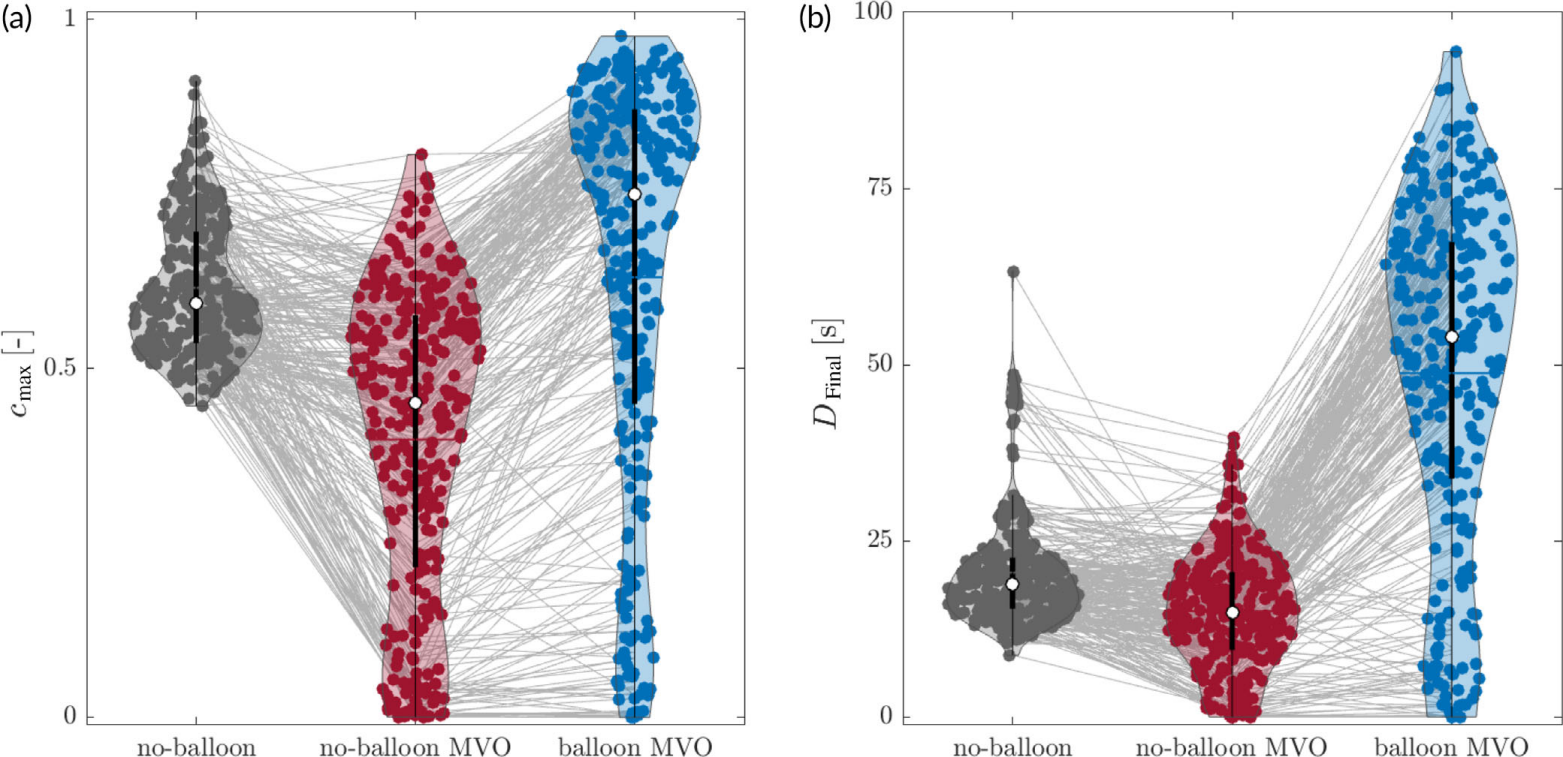
(a) Violin plots of the maximum concentration $c_{\max}$ for the no‐microvascular obstruction (MVO) setting *no‐balloon case* (*dark gray*), *the MVO* setting no‐balloon case (red) and the MVO setting balloon case (blue). Corresponding $c_{\max}$ of the same ROI are connected (solid light gray lines) (b) Violin plots of $D_{\text{final}}$ for the no‐MVO setting *no‐balloon case* (*dark gray*), *the MVO* setting *no‐balloon case* (*red*) *and the MVO* setting balloon case (blue). Corresponding $D_{\text{final}}$ of the same ROI are connected (solid light gray lines).

Figure 5a shows violin plots^20^ of the maximum drug concentration $c_{\max}$ for all ROIs in three different experimental settings: *no‐balloon* experiments without MT/MVO (control experiments), *no‐balloon* experiments with MT/MVO, and corresponding *balloon* experiments with the same MT/MVO setting. Whereas the concentration is mostly above 50%, in the control experiments (*no‐balloon*, *no‐MVO*), the presence of MT (*no‐balloon*, *MVO*) led to the distribution of $c_{\max}$ over a broader range. In general, $c_{\max}$ was lower in most ROIs, and many MT had *c*_max_ < 20%. This corresponds to the group of MT in Figure 4c that were classified as *no‐contact* with dye. The cumulated dose $D_{\text{final}}$ (Figure 5b) follows the same findings (further data are provided in Table 1).

**TABLE 1:** Statistical values: 25%‐Quartile Q1, median Q2, 75%‐quartile Q3, mean for $c_{\max}$, and $D_{\text{final}}$.

		$c_{\max}$ [−]				$D_{\text{final}}$ [s]			
Experiment		Q1	Q2	Q3	$\bar{c_{\max}}$	Q1	Q2	Q3	$\bar{D_{\text{final}}}$
nb	nm	0.54	0.59	0.70	0.61	15.5	18.9	22.5	20.5
nb	m	0.22	0.45	0.57	0.40	09.6	14.8	20.5	15.0
b	m	0.45	0.75	0.87	0.63	33.9	54.0	67.4	48.8

*Note*: For no‐balloon (nb), balloon (b), no‐MVO (nm), and MVO (m).

Abbreviation: MVO, microvascular obstruction.

### 2.5 Proximal balloon inflation and controlled flow drug delivery increase drug concentration in target vessels by 57%

For the *balloon* case with the *MVO* setting (blue in Figure 5a), mean $c_{\max}$ increased by 57% compared with the *no‐balloon* case with *MVO*. However, some of the lowest $c_{\max}$ remained at their low level even with the inflated balloon. Note that the median of $c_{\max}$ for the *balloon* case with *MVO* was higher than in the *no‐balloon* case without *MVO*. The $D_{\text{final}}$ values (Figure 5b) exhibit the same characteristics as $c_{\max}$ (more details in Table 1).

## 3 DISCUSSION

### 3.1 Myocardial MVO with distal embolization could be modeled in a hybrid microchip model

Figures 2 and 4b show that most MT got stuck in channels with diameters between 220 and 50 μm corresponding to channels of orders 6 and 5. This is in line with Schwartz et al.^4^ who found that 89% of MT causing MVO have been found in vessels of diameter <120μm and 54% in vessels >40 μm.

Many MT were found at bifurcations (76%), which may lead to complex configurations where an MT may, for example, fully occlude one branch distal of the bifurcation, whereas the other branch may remain open. This configuration was classified as *semiocclusive* (29% of all cases). This may be relevant for treatment with antithrombotic or thrombolytic drugs because the semipermeable character allows the drug to reach its target easier.

The presence of MT in the microchip leads to degraded perfusion of the network, as it would also be expected for MVO. The effect of degraded perfusion due to MVO on drug transport is demonstrated in Figure 5a: In the *no‐balloon* experiments, the mean dye concentration for the *MVO* setting is 34% lower than the mean dye concentration in the control experiments (24% for the median).

For those reasons, the authors believe that the used hybrid in vitro MVO model mimics coronary microvasculature in the presence of small vessel thrombosis close to reality.

### 3.2 Not all microthrombi are reached by drug

Fully occlusive thrombi (44% of all MT) pose a more complex problem for drug delivery because the complete occlusion prevents any flow in the channel such that drug transport by advection is not effective. As seen in Figures 4c and 5, there is a non‐negligible amount of 21% of all MT (*balloon* case), respectively 25% in the *no‐balloon* case, that did not experience direct dye contact with a concentration of at least 20%. Only diffusive transport can bring drugs close to such *no‐contact* MT. Hence, it is important to bring high drug concentrations as close as possible to the MT, to ease diffusion.

However, the effect of *no‐contact* MT may be somewhat exaggerated in the present experimental setup: The used setup compensates for the missing compliance in the solid microchip with resistance‐compliance (RC) elements that are placed distally to the microchip resistance‐compliance elements (see Section 4.1). Therefore, the compliance vanishes if a channel is blocked by an MT. This has a direct and deteriorating impact on the drug delivery, because vascular compliance enables, together with the intramyocardial pump effect, also induced into the system by the RC elements, advective, oscillatory flow also into dead‐ended regions of the vascular network.^16^ As further explained in Ref. 16, stokes flow (Reynolds number below unity) is an important factor influencing the mixing efficiency of advection because Stokes flow is known to be reversible such that oscillatory flow leads to zero net mass transport. Due to the full occlusion and the missing compliance in those fully occluded segment parts, Stokes flow and a resulting flow reversibility of the flow must be assumed. At the same time, this model limitation could be minor, since vascular compliance could also be significantly reduced in heavily or for longer MVO‐affected tissue: it is known that MVO, and inflammatory and vasoconstricting substances often found in plasma after MI, may lead to local reduction of the myocardium's contractile function.^21^, ^22^, ^23^ This suggests that the vessel's compliance is reduced which does not justify approving fully rigid channels but may reduce the described shortcomings of the model to a certain degree.

### 3.3 Proximal balloon occlusion increases maximal concentration

Figure 5a,b shows a relevant increase for $c_{\max}$ and $D_{\text{final}}$ by inflating the balloon during the infusion and applying a balloon‐holding phase. This aligns with the results from Ref. 16, where $c_{\max}$ could be improved by a factor of 2.2–3.2 and $D_{\text{final}}$ by a factor of 4.6–5.2. In the current investigation, the balloon application improves the mean $c_{\max}$ by a factor of 1.58 (median by 1.66), and for $D_{\text{final}}$ by factors of 3.24 (mean) and 3.64 (median). This decrease could be expected since, with the current MVO model and the corresponding ROI, the concentration next to the MT in obstructed network channels is considered. The results from the simpler MVO model from Ref. 16 are based on arbitrarily defined ROI in a disturbed perfused network without any direct association with local MT.

It is essential to point out that the reduced effect of the balloon is mainly due to MT with no contact. For these, mostly *fully occlusive*, MT, the balloon does not seem to improve the situation significantly (compare Figure 4c and Figure 4d, where the values for the *balloon* and the *no‐balloon* case are almost identical). For the *semi‐occlusive* MT; however, the impact of the balloon is strong. The numbers imply that for agents where high $c_{\max}$ and $D_{\text{final}}$ are favorable, a *balloon* procedure may be advantageous for treating patients because the *balloon* case reaches similar or higher values for $c_{\max}$ and $D_{\text{final}}$ than in the *no‐balloon* case.

Nevertheless, a balloon occlusion during dye infusion did not lead to the full contact of the dye with all MT during the simulated MVO treatment. However, most MT get higher $c_{\max}$ and $D_{\text{final}}$. Also, due to the generally higher concentrations in the whole arteriolar tree, the chances that some medication reaches the no‐contact MT are potentially increased. This could already be an improvement for most patients considering the very limited efficiency of today's treatment methods.

### 3.4 Limitations

The presented MVO model is modeling only distal microembolization (by MT injected into the network from proximally). This could, for example, model debris from the primary thrombus in a myocardial infarction treated by PCI. Locally formed MT (e.g., due to the release of prothrombotic factors^23^) may present additional problems and act differently on perfusion. Also, other causes of MVO, like tissue swelling, inflammation, or vasoconstriction, would affect the single vessels differently than the used MVO model.

Further, there is a certain lack of compliance in the rigid microchip. Further elaborations on this topic can be found in Section 3.2.

So far, only one drug infusion protocol with simultaneous balloon occlusion has been tested. The influence of infusion rate or duration of the balloon occlusion should be tested to optimize drug delivery.

To this point, no pharmacokinetics were considered, and higher $c_{\max}$ and $D_{\text{final}}$ were rated as beneficial. A further and important implication of the balloon occlusion and the holding phase is that during this phase no blood flow is present. Importantly, some therapeutic agents act indirectly by activating blood components to dissolve the thrombus, such that $c_{\max}$ may have to remain within a certain range for optimal treatment effect.

Furthermore, this study does not consider the interaction of endothelial (dys‐)function connected to MI and ischemia–reperfusion injury after PCI, as well as the complex interplay of MTs with drug and soluble prothrombotic and vasoconstricting substances in plasma.^23^ A continued investigation considering pharmacokinetics and other aspects such as the influence of prothrombotic factors and endothelial damage and dysfunction in the microcirculation, is important.

## 4 MATERIALS AND METHODS

### 4.1 Coronary model

**FIGURE 6:**
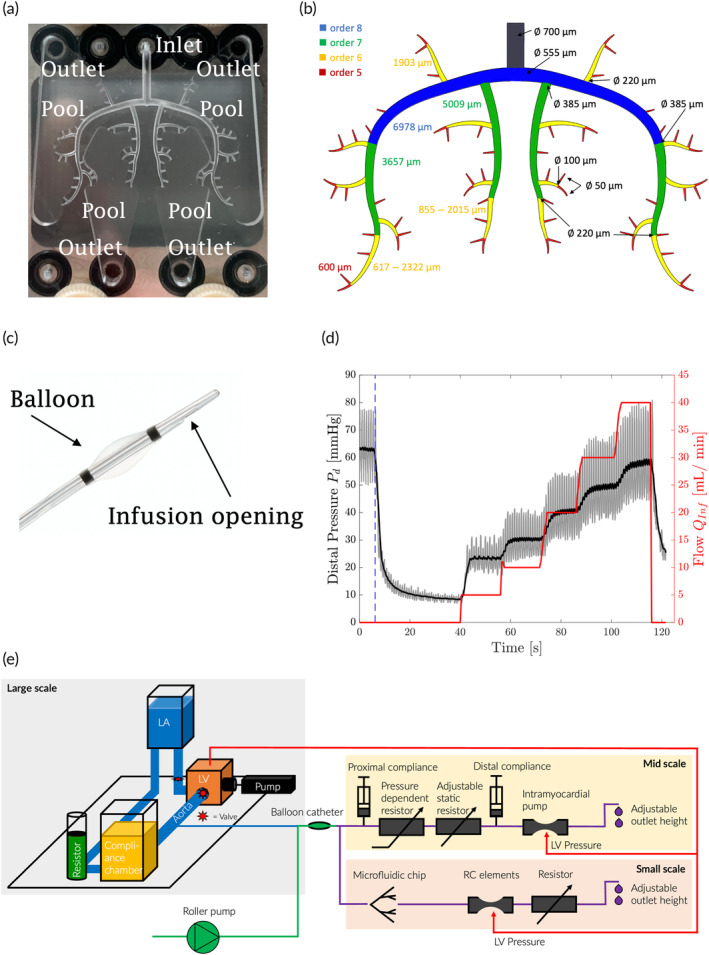
(a) Top view of the microfluidic chip. (b) Schematic of the microchip design with segment lengths (left side) and segment diameters (right side). (c) Occlusion‐infusion catheter with a balloon to block a coronary artery proximally, a lumen for controlled fluid infusion and a pressure wire for distal pressure measurement. (d) Distal coronary pressure (gray) with mean pressure (black) during a diagnostic occlusion‐infusion sequence (OIS; $Q_{\mathrm{Inf}}$ shown in red, timepoint of balloon inflation marked with blue‐dashed line) performed on a pig with microvascular obstruction. (e) Schematic of the left‐heart mock loop and the coronary circulation model with impedance elements and microchip (adapted Figures from Ref. 16).

The multiscale benchtop model (Figure 6e) of this study was first described in Thirugnanasambandam et al.^17^ and later extended by a microfluidic chip^16^ (Figure 6a,b). A left‐heart mock loop was used to model the large‐scale hemodynamic. It comprised a computer‐controlled piston pump, a ventricular chamber, mechanical heart valves in the mitral and aortic position, a silicone aortic root phantom, a compliance chamber, a static resistor, and a left atrium (Jahren et al.^24^).

A model of the LAD coronary artery represented the mid‐scale. It was connected to one of the two ostia in the aortic root phantom of the left‐heart mock loop. This mid‐scale model consisted of several hydraulic resistance and impedance elements reproducing a classical coronary waveform with peak coronary flow during diastole.^18^

A fluidic microchip was used to model the coronary circulation's small scale and to investigate perfusion in the coronary arteriolar tree. This glass microchip featured SLE circular channels modeling four generations of vessels in the arteriolar tree with diameters from 555 down to 50μm. Based on anatomical data from Kassab,^18^ the microchip channels correspond to ~2% of the microvascular tree fed by the LAD. More information about the chip's layout, channel size, and fabrication method can be found in Ref. 16.

The microchip was mounted on a titanium plate with different channels and connections to ensure better handling. In the middle of this plate was a window to provide optical access for a backlight (LED Microscopy backlight, 50 mm, 9 W, BoliOptics, California, USA) placed beneath the plate. A camera (Body: Fastcam Mini AX 100, Photron; Lenses: F2.8/100mm Macro Lens, Samyang; Intermediate Ring set, Walimex) was mounted on top of the microchip to record the dye distribution in the microchip. Flow and compliance tuners (RC elements) were added distal to the microchip to compensate for the missing compliance in the rigid microchip.^16^

A multi‐lumen catheter was placed before the bifurcation of the LAD model and the plate with the microchip (see Figure 6c).

### 4.2 Working fluids

A viscosity‐matched blood‐mimicking fluid (BMF) at room temperature was used as a working fluid. It was a mixture of 60% distilled water and 40% Glycerol (ReagentPlus®, ≥99.0% (GC), Sigma‐Aldrich, Buchs, Switzerland) by weight.^25^ This BMF was filled in the left‐heart mock loop and the coronary model.

A diluted 0.1% crystal violet solution (Crystal Violet, 1% aqueous solution, Sigma Aldrich, diluted with distilled water to 0.1%) was used to model the infused drug. The viscosity of the dye solution is waterlike, as is the case for many infused drugs. During the experiments, this dye solution was infused over the occlusion‐infusion catheter into the LAD model proximal to the microchip.

### 4.3 Controlled flow infusion

The CoFI system (CorFlow Therapeutics AG, Baar, Switzerland) can measure pressure‐flow relations in the coronary circulation and locally infuse drugs. It is based on a custom multilumen rapid‐exchange occlusion‐infusion balloon catheter (Figure 6c). The multilumen catheter can occlude an epicardial blood vessel by inflating a balloon. A pressure‐sensing guidewire is used to measure the blood pressure distal to the balloon (OptoWire Deux, OpSens, Québec, Canada). The third lumen is used for the simultaneous infusion of fluids distal to the balloon. The controlled infusion is driven by a roller pump (LiveTec LiveCool Infusion Pump, livetec Ingenieurbüro GmbH, Lörrach, Germany).

In this study, flow‐pressure measurements with the CoFI system were used to tune the coronary model to data acquired previously with the same system in a reference animal. After tuning the model, the CoFI system was used to infuse the dye solution into the coronary model at controlled flow rates while the balloon was either inflated or deflated.

### 4.4 Microthrombi

MT used in this study were created within the framework of a different research project for which the animal study was approved by the local Committee for Animal Experimental Research (Cantonal Veterinary Office Zurich, Switzerland) under the license number ZH213/2019. While under general anesthesia, a midline incision of ~15 cm was performed on the ventral side of the neck with the animal in dorsal recumbence. The right carotid artery was bluntly dissected over a section of ~10 cm. Using a standard haemostatic clamp, the distal half of the prepared carotid artery was crushed at multiple locations with a distance of 0.5 cm for 5 s. A proximal ligature was used to reduce blood flow during thrombus formation. Thirty minutes postcrush, the carotid artery was examined for the progress of thrombus formation by direct supravascular ultrasound, and a second series of crushes was performed as described above if no thrombus formation was detected. Sixty minutes following vascular crush, the carotid artery was ligated and excised. The thrombus material formed within the carotid artery was used to create MT of about 200 μm in a manual shredding procedure. As reported in Cesarovic et al.,^26^ the MTs show a heterogeneous structure with regions of high corpuscular density containing activated platelets, erythrocytes, and only occasional leukocytes adjacent to loosely interconnected fibrin‐mesh and a tight fibrin‐layer on the surface.

The MT were cold (4°C) stored in 0.9% NaCl in aliquots of 20 pieces until their use in the microchips, but at maximum for 48 h.

### 4.5 Experimental protocol

The left‐heart mock loop was filled with BMF and tuned to an aortic blood pressure of ~52/82 mmHg at a heart rate of 69 bpm to model a pig with MVO.^17^ The coronary model was filled with distilled water and tuned to match the flow‐pressure curve (Figure 6d) obtained from an OIS experiment in a pig. Details on the procedure for model tuning are given in Ref. 17.

Next, the flow through the microchip was adjusted to 2% of the total flow rate through the coronary model (in accordance with the relative volume of the modeled microvasculature). To this end, the balloon was inflated and the infusion rate $Q_{\mathrm{Inf}}$ was set to 30 mL/min. The static resistor distal to the microchip (Figure 6e) was then successively closed until the flow through the microchip was 0.6 mL/min.

After that, the microchip was dismounted and a syringe filled with ~20 MT suspended in 0.9% NaCl solution was placed at the inlet of the microchip and the full content of the syringe was injected into the microchip. MT that were not flushed into the microchip in this process (e.g., due to spilling) were discarded. Therefore, not all experiments had the same amount of MT. After filling the microchip with MT, it was mounted again in the setup and the rest of the model was filled with BMF. Finally, the reservoir of the CoFI system and the catheter were filled with the dye solution.

Two drug infusion protocols were studied: In the *no‐balloon* case, 15 mL dye were infused through the occlusion‐infusion catheter over a period of 30 s (infusion phase). The balloon remained deflated throughout these experiments. The second case, called *balloon*, started with the inflation of the balloon to occlude the vessel proximal to the microchip. This was followed by the same infusion phase as in the no‐balloon case (15 mL dye in 30 s) while keeping the balloon inflated. The balloon inflation was maintained for 60 s after the end of the infusion (balloon‐holding phase). After that, the balloon was deflated (total balloon occlusion time 30 + 60 s).

This infusion volume of 15 mL corresponds to one third of the recommended dose (systemic application) for the treatment of a 90 kg heavy person with AGGRASTAT (Inf sol 1.25 mg/250 mL), a (GP)‐IIb/IIIa‐receptor antagonist). Only one third was used based on the assumption that less drug is needed with the intracoronary application than in a systemic approach.

**FIGURE 7:**
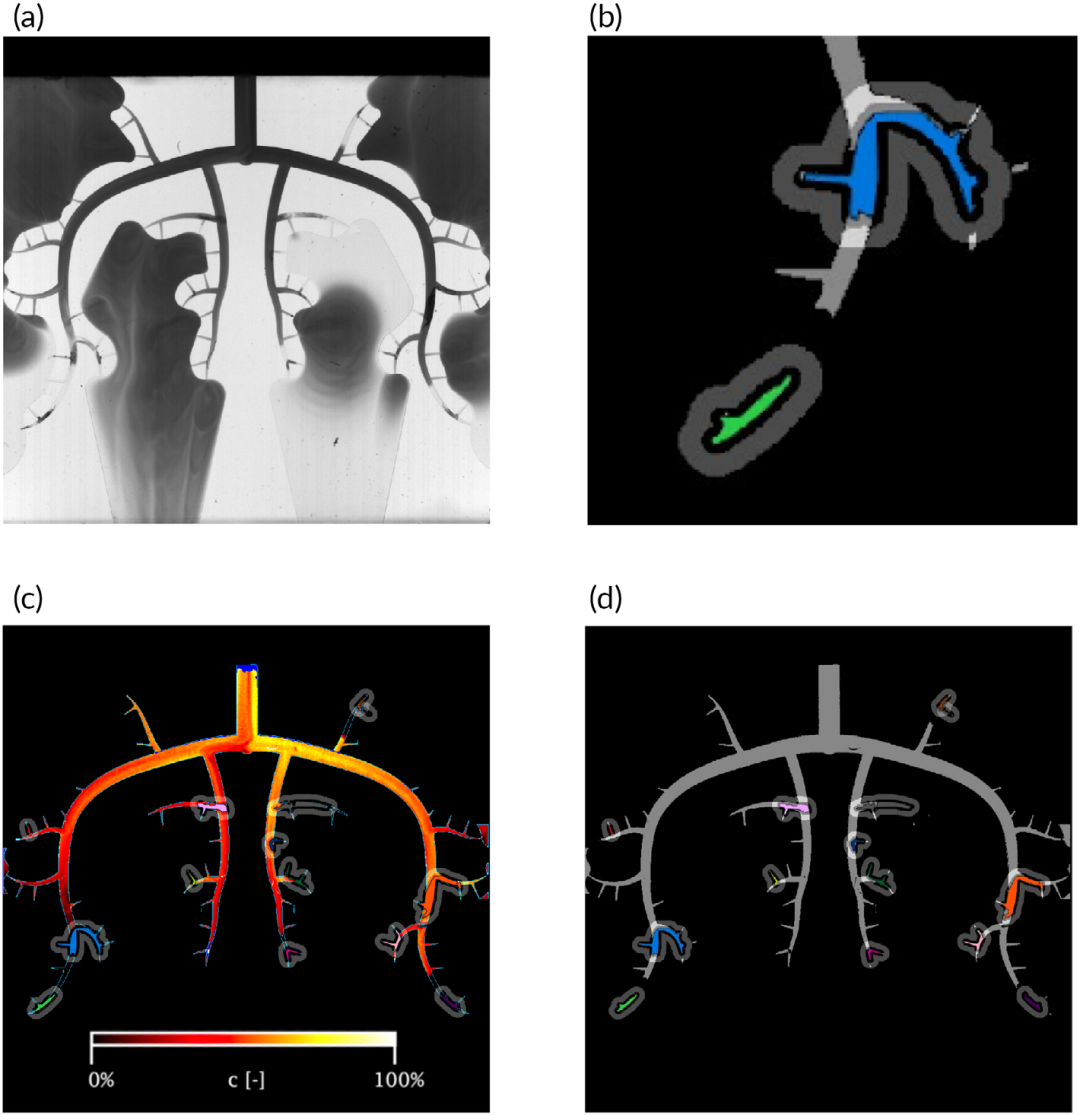
(a) Microchip partially perfused with dye. (b) Detail view of two microthrombi (MT; colored area) with the surrounding annulus‐shaped ROIs (shaded area). (c) Heatmap with calculated dye concentration after postprocessing algorithm with the MT (different colors) and the surrounding region of interest in gray. (d) 20%‐contact map. The most distal ends have not seen concentrations over 20%.

The illuminated microchip was recorded during the experiments by a camera with 10 Hz, and illumination and shutter were configured to use the illumination range of the camera as widely as possible (Figure 7a). The camera started recording 10 s before the infusion started and recorded 180 s in total.

In total, 20 different MT settings (*MVO* setting) were studied. For each MVO setting, a *balloon* and a *no‐balloon* experiment were performed. A balloon and a no‐balloon case were recorded as a reference case before injecting MT (*no‐MVO* setting).

### 4.6 Data analysis

A custom‐made postprocessing algorithm extracted the area containing the channels (Figure 7c) from the recorded video frames of the microchip (Figure 7a).

A previous calibration procedure (see Appendix S1) was used to obtain calibration curves for each pixel, which allowed determining a time‐resolved dye concentration for the whole channel structure (Figure 7c).

Next, the algorithm automatically detected the position and shape of the MT (Figure 7b) which was visually checked afterwards. To define regions of interest (ROI) in the vicinity of each thrombus, the labeled thrombus areas were enlarged in all directions by 16 pixels (ca. 240 μm). From these enlarged areas, the area of thrombi enlarged by 5 pixels (ca. 75 μm) was removed. This resulted in an annulus (width ca. 165 μm) around each microthrombus, which was defined as the ROI of the corresponding microthrombus. The distance of 16 pixels from the thrombi corresponds to the diffusion length that was found to reduce a starting concentration of 20% by a factor of 4 during 1 min (= the balloon‐holding phase). Therefore, this was assumed to be the minimal distance a drug needs to come close to a thrombus to have some effect. The gap between ROI and thrombus of 5 pixels was chosen such that small movements of the MT during the experiments did not interfere with the ROI.

The mean concentration over all pixels of each ROI was calculated for each video frame. To remove pulsatility in the signal, low‐pass filtering with a moving average filter (width of one heartbeat) was applied. This filtered signal was defined as the dye concentration $c\left(t\right)$, ranging from 0% to 100%.

Because the time a drug remains next to a thrombus may also be important for the drug efficacy, the cumulated dose $D\left(t\right)$ was determined as the integral of the concentration *c* over time,

$$D\left(t\right)=\int_{0}^{t}c\left(t^{\prime }\right)dt^{\prime }.$$

For this study, all aspects of pharmacokinetics were neglected which may be very specific to particular drugs. It was simply assumed that higher concentrations c and higher cumulated doses D indicate better efficacy of the drug delivery.

For the comparison of the different experiments, the maximum concentration $c_{\max}$ and the final cumulative drug dose $D_{\text{final}}$ were defined as

$$c_{\max}=\max_{t}c\left(t\right)$$

$$D_{\text{final}}=D\left(t=180s\right).$$

For further analysis of the MVO settings, the MT were categorized as follows:

1. *Size and location*: for every thrombus, the number of segments of the arteriolar tree in which the thrombus resided and the order of these segments were determined. Here, a *segment* refers to the vessel segment between two successive bifurcations. A segment containing a part of a microthrombus was called a *thrombus segment*.
2. *Segment occlusitvity*: Segments were marked as *occlusive* if all pixels in the distal segments had dye concentrations *c*(*t*) of <20% at all times during the experiment. Otherwise, the segment was marked as *nonocclusive*.
3. *Thrombus occlusitvity*: Depending on the *occlusitvity* of the thrombus segments, a microthrombus could be either *nonocclusive* (all thrombus segments were nonocclusive), *fully occlusive* (all thrombus segments were occlusive), or *semiocclusive* (otherwise).
4. *Contact*: Every microthrombus was categorized according to the concentration level in the ROI. They were considered to be in *contact* if $c\left(t\right)\geq 20\%$ at the edge of the thrombus. They were in the *diffusion range* if $c\left(t\right)\geq 20\%$ in the annulus, or they were in *no‐contact* if $c\left(t\right)<20\%$ in the whole ROI. An example of a 20%‐contact map is shown in Figure 7c.

## 5 CONCLUSION

These experiments show an increase in the maximal concentration $c_{\max}$ as well as for the final cumulated dose $D_{\text{final}}$ of an infused dye when simultaneously occluding the coronary vessel proximally with a balloon during intracoronary dye infusion (representing drug) into a vessel network with distal embolisation. The local $c_{\max}$ and $D_{\text{final}}$ around the real porcine MT could be increased by 58% (mean), respectively 224% (mean). Further investigations, considering pharmatokinetic effects, are necessary and could lead to a real improvement in future MVO treatment methods.

## AUTHOR CONTRIBUTIONS

**Yannick Rösch:** Conceptualization (equal); formal analysis (equal); investigation (equal); writing—original draft (equal); writing—review and editing (equal). **Thorald Stolte:** Investigation (equal); writing—review and editing (equal). **Miriam Weisskopf:** Investigation (equal); writing—review and editing (equal). **Sabrina Frey:** Conceptualization (equal); writing—review and editing (equal). **Rob Schwartz:** Conceptualization (equal); writing—review and editing (equal). **Nikola Cesarovic:** Conceptualization (equal); investigation (equal); writing—review and editing (equal). **Dominik Obrist:** Conceptualization (equal); writing—review and editing (equal).

## FUNDING INFORMATION

This study was financed over the (grants 25390.2PFLS‐LS, 31010.1IP‐LS, and 104.947 IP‐LS) from the Innosuisse – Schweizerische Agentur für Innovationsförderung.

## CONFLICT OF INTEREST STATEMENT

SF and RSS own stock and/or stock options in CorFlow Therapeutics AG. SF is an employee of CorFlow Therapeutics AG. SF, RSS and DO have a pending patent application for a microfluidic coronary circulation model. CorFlow Therapeutics AG partially funded the work of YR. YR, TS, MW, and NC and were provided free material (e.g., Occlusion‐infusion catheter) from CorFlow Therapeutics AG.

### PEER REVIEW

The peer review history for this article is available at https://www.webofscience.com/api/gateway/wos/peer-review/10.1002/btm2.10631.

## Supporting information


**FIGURE S1:** Distribution of projected area of MTs.

## Data Availability

The original data that support the findings of this study are available from the corresponding author upon reasonable request.
