# Supplementary material for: Efficacy of catheter‐based drug delivery in a hybrid in vitro model of cardiac microvascular obstruction with porcine microthrombi
Source: Bioeng Transl Med. 2023 Dec 8;9(2):e10631. doi: 10.1002/btm2.10631 (PMC10905539; doi:10.1002/btm2.10631)
Supplement: Supplementary file 1 — FIGURE S1: Distribution of projected area of MTs. [file BTM2-9-e10631-s001.docx]

# Appendix / Supplementary material

## Calibration

For the optical analysis of the dye concentration in the microchip, the experiment was calibrated on every experimental day. To this end, the microchip was mounted in the set-up, and backlight illumination was adjusted so that the “white” areas of the empty microchip had RGB values around $240 - 250$. The microchip was then uniformly filled with BMF for the $0 \%$ dye, mixtures of BMF and dye at concentrations of $10, 20, 40, 60, 80, 100$, and a $110 \%$dye without BMF, and then recorded with the camera. The data point at $110 \%$ was added to have one extra calibration point outside the used range. A concentration of $110 \%$ could be achieved because the $100 \%$ solution was already a dilution of the used dye. All video frames were corrected for brightness (compared to the first frame), rotation and translation (compared to a master alignment frame).

Between the different concentrations, the chip was flushed with BMF.

In the postprocessing of the video frame, a calibration curve was calculated for every pixel in the detected network structure (Figure 7). To this end, the parameters a, b, c of the function

$c\left( I \right)= \frac{1}{a*\sqrt{I}+b}+c$

were optimised to minimise the root-mean-square error (RMSE),

$$RMSE = \sqrt{\frac{1}{N} \sum_{j=1}^{N} \left[ c_{j}-c\left( I_{j} \right) \right]^{2}}$$

where $I_{j}$ was the mean pixel intensity (averaged over ten frames) at the known concentration $c_{j}$ and $N = 8$. Pixels for which no satisfactory calibration curve could be established were excluded from the analysis.

**Figure S1**


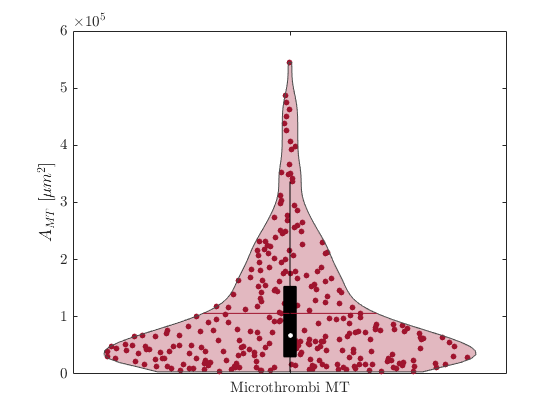


**Figure S1:** Distribution of projected area of MTs.
